# Letter to Editor on Dual Anti-Epileptics Induced Stevens-Johnson Syndrome: A Case Report

**DOI:** 10.31729/jnma.8661

**Published:** 2024-07-31

**Authors:** Vikash Paudel

**Affiliations:** 1 Department of Dermatology and Venereology, Patan Academy of Health Sciences, Lagankhel, Lalitpur, Nepal

**Dear Editor,**

I have read the case report entitled "Dual Antiepileptics Induced Stevens-Johnson Syndrome: A Case Report" by Nakarmi et al., published in Journal of Nepal Medical Association 2020;58(230):801-4.^1^ I would like to take this opportunity to congratulate the authors and the supporting staff for successfully managing the life-threatening severe cutaneous adverse drug reaction caused by anti-epileptics and would like to make some comments regarding this.

Stevens-Johnsons syndrome (SJS) and toxic epidermal necrolysis (TEN) are severe cutaneous adverse drug reactions. These are more common with aromatic antiepileptic drugs; however, they can also occur with other antiepileptic drugs which can cross-react with each other and one may increase the chance of severe drug reactions if combined.^2^ The rash predictor study of 15 potential antiepileptic drugs had revealed that the highest rash rates occurred with phenytoin (5.9%), lamotrigine (4.8%) and carbamazepine (3.7%) whereas the lowest rash rates occurred with felbamate, primidone, topiramate (all <1%), levetiracetam (0.6%), gabapentin (0.3%), and valproate (0.7%).^3^

In the report, it has been mentioned that the patient suffered from SJS due to dual antiepileptics i.e., phenytoin and sodium valproate after 26 days of exposure. The patient was admitted for some seizure disorders who was given phenytoin, sodium valproate, and clobazam during the hospital stay and continued on discharge.

I would like to comment on three important factors regarding the management and diagnosis of SJS here. The first one is that, if the lesion in the patient was a suspicion of SJS due to some antiepileptic drugs, all antiepileptic drugs including clobazam should have been stopped, because there are reports of SJS even with clobazam.^4-6^ It is rightly said that all drugs except life-saving or emergency drugs should be stopped in suspicion of minor SJS since it could be converted to TEN which could be more fatal.^2,7^ Since, clobazam is not a life-saving drug it should also have been stopped. Besides, co-administration of one antiepileptic drug has been shown to significantly increase the plasma concentrations of other antiepileptic drugs and the risk of a potentially serious and life-threatening rash. It's said that when clobazam is added to a therapy regimen that includes valproic acid, the patient should be closely monitored for possible adverse drug reactions caused by elevated valproic acid serum concentrations, and monitoring of valproate serum levels should be considered.^8^

Secondly, since phenytoin is one of the commonest antiepileptic drugs causing SJS, valproate might not be the cause of SJS here, since we cannot prove whether the combination or a single drug was responsible. There is no doubt about valproate being the potential cause of SJS or TEN singly as well.^3^

Thirdly, the drug-drug interaction between phenytoin, valproate, and clobazam could have contributed to the development of SJS, which we cannot establish the causality.

Since three antiepileptic drugs were used and SJS followed it, on the basis of temporal association, triple antiepileptic drug-induced SJS could have been the better title.

Moving forward, it's crucial for healthcare practitioners to adhere to standard guidelines and promptly withdraw all implicated drugs except life-saving ones when managing severe cutaneous adverse drug reactions like SJS and TEN. Further research and vigilance in clinical practice are essential to better understand and prevent such life-threatening reactions in patients receiving antiepileptic therapy.
